# The Polycomb Group Protein Pcgf1 Is Dispensable in Zebrafish but Involved in Early Growth and Aging

**DOI:** 10.1371/journal.pone.0158700

**Published:** 2016-07-21

**Authors:** Barbara Dupret, Pamela Völkel, Xuefen Le Bourhis, Pierre-Olivier Angrand

**Affiliations:** 1 Cell Plasticity & Cancer, Inserm U908 / University of Lille, Lille, France; 2 CNRS, Lille, France; University of Sheffield, UNITED KINGDOM

## Abstract

Polycomb Repressive Complex (PRC) 1 regulates the control of gene expression programs via chromatin structure reorganization. Through mutual exclusion, different PCGF members generate a variety of PRC1 complexes with potentially distinct cellular functions. In this context, the molecular function of each of the PCGF family members remains elusive. The study of PCGF family member expression in zebrafish development and during caudal fin regeneration reveals that the zebrafish *pcgf* genes are subjected to different regulations and that all PRC1 complexes in terms of Pcgf subunit composition are not always present in the same tissues. To unveil the function of Pcgf1 in zebrafish, a mutant line was generated using the TALEN technology. Mutant *pcgf1*^*-/-*^ fish are viable and fertile, but the growth rate at early developmental stages is reduced in absence of *pcgf1* gene function and a significant number of *pcgf1*^*-/-*^ fish show signs of premature aging. This first vertebrate model lacking Pcgf1 function shows that this Polycomb Group protein is involved in cell proliferation during early embryogenesis and establishes a link between epigenetics and aging.

## Introduction

In eukaryotes post-translational modifications of histone proteins play a crucial role in chromatin organization and in the control of gene expression programs. Polycomb Group (PcG) proteins are part of the enzymatic machineries involved in these histone modifications [1]. PcG proteins interact to form two major multiprotein complexes, Polycomb Repressive Complex 1 (PRC1) and PRC2 which catalyze two post-translational histone modifications involved in chromatin compaction and gene silencing. PRC1 is responsible for the monoubiquitinylation of histone H2A at lysine 119 (H2AK119ub1) whereas PRC2 trimethylates lysine 27 of histone H3 (H3K27me3) allowing the recruitment of PRC1 [2–5].

In *Drosophila*, core PRC1 is composed of Polycomb (Pc), a chromodomain-containing protein binding to H3K27me3 marks [6, 7], Sex combs extra (Sce) which is responsible for H2A ubiquitinylation [5], Posterior sex combs (Psc) able to compact chromatin *in vitro* [8], and the poorly characterized protein Polyhomeotic (Ph). In contrast, vertebrate PRC1 complexes are very heterogeneous since each of the *Drosophila* subunits is encoded by several orthologs that can associate in a combinatorial fashion. Mammalian genomes contain five orthologs for Pc (CBX2, 4, 6, 7 and 8), two orthologs for Sce (RING1 and RNF2), five Psc genes (BMI1, PCGF1, 2, 3, 5 and 6) and three Ph orthologs (PHC1, 2 and 3). A number of reports have shown that multiple PRC1 complexes, distinct in their subunits composition due to combinatorial permutations, exist in human cells [9–16]. To complicate matters further, several PRC1 subunits could also associate to form non-canonical PRC1 complexes [13, 14, 17, 18]. In addition, mice deficient for individual PRC1 components show distinct phenotypes suggesting that different PRC1 complexes might have at least some non-redundant functions [19–26]. Thus, understanding the individual role of the distinct isoforms of the PRC1 protein complexes remains an objective towards the deciphering of the Polycomb-mediated repression in vertebrates.

The zebrafish (*Danio rerio*) is a widely used vertebrate model for studying development and morphogenesis. Owing to external fertilization and optical transparency of the embryos, early development of zebrafish can be easily followed. Furthermore, the recent emergence of powerful genome-editing technologies, such as the Transcription Activator-Like Effector Nucleases (TALENs) and the Clustered Regularly Interspaced Short Palindromic Repeats / CRISPR-associated System (CRISPR/Cas9) applied to zebrafish, allows rapid gene function studies in this organism [27–32].

Here, we report on the expression of the Psc family members during zebrafish development and during caudal fin regeneration. We provide evidences that the different genes are differentially expressed suggesting that distinct PRC1 variations occur during zebrafish development and caudal fin regeneration. Using the TALEN technology, we generated a *pcgf1* loss-of-function zebrafish line. This line is viable and fertile indicating that Pcgf1 is dispensable in zebrafish. However, we show that zebrafish growth at early developmental stages is reduced in the absence of *pcgf1* gene function and about 35% of *pcgf1*^*-/-*^ fish show signs of premature aging.

## Materials and Methods

### Ethical Statement

The zebrafish experiments described in this study were conducted according to the French and European Union guidelines for the handling of laboratory animals. The experimental procedures carried out on zebrafish were reviewed and approved by the local Ethics Committee from the Animal Care Facility of the University of Lille. None of the animals utilized in this work became ill or died before the experimental endpoint. At the end of the experiment, fish older than 8 dpf were humanely euthanized by immersion in an overdose of tricaine methane sulfonate (MS-222, 300 mg/L) for at least 10 minutes, whereas younger fish were immobilized by submersion in ice water (5 parts ice/1 part water, 0–4°C) for at least 20 minutes to ensure death by hypoxia.

### Zebrafish maintenance and embryo preparation

Zebrafish (TU strain) were maintained at 27.5°C in a 14/10h light/dark cycle. The evening before spawning, males and females were separated into individual tanks. Spontaneous spawning occurred when the light turned on and embryos or larvae were collected and staged according to Kimmel et al. [33]. The chorions were removed from embryos by the action of 1% pronase (Sigma) for 1 min, and embryos or larvae were fixed overnight in 4% paraformaldehyde in PBS (phosphate-buffered saline, Invitrogen), dehydrated in 100% methanol and kept at -20°C.

For caudal fin amputation, 3 to 6 month-old zebrafish were anaesthetized with MS-222 (tricaine, ethyl 3-aminobenzoate methanesulphonate, 0.168 mg/mL; Sigma-Aldrich) and approximately two-thirds of the fin was cut with a blade. After fin amputation, the fishes were allowed to regenerate their fin in the aquarium until 4 days at 27.5°C. The blastemal starts to form at approximately 24-hours post amputation (hpa) and the amputated fins have been fully restored at around 14-days post amputation (dpa).

### Genotype analyses

Three day-old embryos or pieces of caudal fin were incubated in 10 μL of PCR extraction buffer (10 mM Tris-HCl pH8.0, 2 mM EDTA, 0.2% Triton X-100, 100 μg/mL Proteinase K) and placed at 50°C for 4 hours prior proteinase K inactivation at 95°C for 5 min. Genotype analysis was performed by PCR on 2.5 μL of samples using the primer set TAL_pcgf1_5' (5’-GAACACAATGAACACAATGGC-3’) and TAL_pcgf1_3' (5’-GGACAATACTTGCTGGTCTGG-3’) followed by PCR product digestion with the ClaI restriction enzyme. Sequence determination (GATC-Biotech, Germany) was performed after cloning of the PCR products into the vector pCR-XL-TOPO (Invitrogen) according to the manufacturer’s instructions.

### In situ hybridization

Antisense RNA probes were synthesized with the DIG RNA Labeling Kit (SP6/T7) (Roche, 11175025910), according to the manufacturer’s instructions from 1 μg of linearized plasmid DNA. The following IMAGE cDNA clones purchased at imaGenes GmbH (Berlin), were used: *pcgf1*, cDNA clone MGC:162519 IMAGE:7165875; *bmi1a*, cDNA clone MGC:56403 IMAGE:5605189; *bmi1b*, cDNA clone MGC:63927 IMAGE:6790897; *pcgf5a*, cDNA clone MGC:136815 IMAGE:7258679; *pcgf5b*, cDNA clone MGC:194668 IMAGE:9039003; *pcgf6*, cDNA clone MGC:162242 IMAGE:8745325. *In situ* hybridization was performed as described by Thisse and Thisse [34]. Briefly, the fixed embryos were rehydrated and permeabilized with 10 μg/mL proteinase K for 30 sec (0.75 to 8 hpf embryos), 1 min (12 and 16 hpf embryos) or 10 min (24 hpf embryos) at room temperature. Ten to 15 embryos from each time points were hybridized with digoxigenin-labeled antisense RNA probes at 70°C. After extensive washing, the probes were detected with anti-digoxigenin-AP Fab fragment (Roche Diagnostics, 1093274, diluted at 1:10,000), followed by staining with BCIP/NBT (5-bromo-4-chloro-3-indolyl-phosphate/nitro blue tetrazolium) alkaline phosphate substrate.

The embryos were imaged using a Leica MZ10F stereomicroscope equipped with a Leica DFC295 digital camera.

Regenerating fins were fixed in 4% paraformaldehyde overnight, embedded in paraffin, and cut into 7-μm-thick sections. These were mounted on sylanated glass slides, deparaffinated and rehydrated before performing *in situ* hybridization on slides.

### TALEN design and assembly

The *pcgf1* TALEN target site was selected using the online TAL Effector-Nucleotide Targeter tool (https://tale-nt.cac.cornell.edu/; [35]) in exon 2 with the following parameters: (i) spacer length of 14–17 bp, (ii) repeat array length of 16–18 bp, (iii) each binding site was anchored by a preceding T base in position ‘‘0” as has been shown to be optimal for naturally occurring TAL proteins [36, 37], (iv) presence of a restriction site (ClaI) within the spacer sequence for screening and genotyping purposes.

Pcgf1-specific TALEN constructs were engineered using the TALEN Golden Gate assembly system described by Cermak et al., [38]. The TALEN expression backbones, pCS2TAL3DD and pCS2TAL3RR [27], and the plasmids providing repeat variable diresidues (RVD) [38] for Golden Gate Cloning were obtained from Addgene.

### mRNA injection into zebrafish embryos

Capped mRNAs were synthetized using the SP6 mMESSAGE mMACHINE kit (Ambion) from linearized plasmid templates. mRNAs (50–100 pg) were injected into 1-cell zebrafish embryos using a FemtoJet microinjector (Eppendorf).

### Histone extraction and western blot analysis

Histone extracts were prepared by lysis of 5 embryos per tube in PBS containing 0.5% Triton X-100, 2 mM phenyl-methylsulfonyl fluorid (PMSF), 0.02% NaN_3_, 10 min on ice. After centrifugation, the pellet was resuspended in 0.2 N HCl and core histones extracted overnight at 4°C while rocking. Samples were centrifuged and the supernatant containing the histones was stored at -20°C.

For Western blotting, protein samples in SDS loading buffer were electrophoresed on 4–12% Bis-Tris gels (NuPAGE, Invitrogen) and transferred to nitrocellulose membranes using the iBlot® Dry Blotting System (Invitrogen). Efficiency of the transfer is verified by immersing the blotted membrane into a Ponceau S staining Solution (0.1% Ponceau S in 5% acetic acid, Sigma). The membranes were then destained and blocked in 5% milk powder in PBS-T (1x PBS with 0.1% Tween20) for 1 hour at room temperature, incubated for the same time with the primary antibody in 5% milk in PBS-T, and washed three times 10 min in PBS-T. The membranes were then incubated with the peroxidase-conjugated secondary antibody in 5% milk in PBS-T for 1 hour and afterward washed three times 10 min in PBS-T. Signal was detected using a chemiluminescence substrate (Western Lightning Ultra, PerkinElmer) with a Luminescent Image Analyzer (LAS-4000, Fujifilm).

Primary antibodies used were rabbit anti-H2AK119ub1 (1:500; ABE569, Millipore) and anti-H4 (1:500; ab7311, Abcam). The secondary antibody was a peroxidase conjugated AFFiniPure donkey anti-rabbit antibody (1:10,000; 711-035-152, Jackson ImmunoResearch).

### Whole mount immunohistochemistry

Zebrafish embryos were fixed 2 hours at room temperature in 4% paraformaldehyde, followed by dehydration and storage overnight in methanol at -20°C. Embryos were then digested in PBS containing 0.1% Tween20 and 10 μg/mL proteinase K and blocked in PBS containing 0.1% Tween20 and 2% sheep serum.

The primary antibodies used were mouse anti-MF20 (1:20; Developmental Studies Hybridoma Bank, developed under the auspices of the NICHD and maintained by The University of Iowa, Department of Biology, Iowa City, IA 52242) and rabbit anti-H3S10p (used at 1.33 μg/mL; sc-8656-R, Santa Cruz). The secondary antibodies were a peroxidase conjugated AffiniPure goat anti-mouse (1:500; 115-035-003, Jackson ImmunoResearch) and an Alexa fluor goat anti-rabbit 546 (1:5,000; A-11010, Life Technologies).

### Acridine orange staining

Live dechorionated zebrafish embryos were incubated for 30 minutes at 28°C in E2 medium (15 mM NaCl, 0.5 mM KCl, 0.5 mM MgSO_4_, 0.15 mM KH_2_PO_4_, 0.04 mM Na_2_HPO_4_, 1.3 mM CaCl_2_, 0.7 mM NaHCO_3_) containing 2 μg/mL Acridine Orange (Chroma). Following extensive washes in E2 medium, embryos were anaesthetized with 0.01 mg/mL tricaine (Sigma Aldrich) and imaged with a Leica MZ10F stereomicroscope equipped with a Leica DFC295 digital camera.

### Alcian blue–Alizarin red staining

Zebrafish embryos and larvae were fixed 2 hours at room temperature in 4% paraformaldehyde and dehydrated 10 min in 50% ethanol. Cartilages were stained in 0.02% Alcian blue (Sigma Aldrich), 60 mM MgCl_2_, 70% ethanol, overnight at room temperature. Bones were then stained in 0.1% Alzarin red (Sigma Aldrich) 24 hours at room temperature. Pigments were bleached by a 2-hours incubation in water containing 1% KOH, 3% H_2_O_2_. Embryos and larvae were digested with 0.05% trypsin (Sigma Aldrich) until tissue disappeared (1 to 4 hours depending on the developmental stage) and fixed in 95% ethanol before storage in 70% glycerol.

### Senescence-associated β-galactosidase assay

Adult zebrafish were anaesthetized in MS-222 (0.168 mg/mL) and placed into ice-cold fish water. The fish were washed twice in PBS pH7.4, and fixed for 3 days in 4% paraformaldehyde with PBS at 4°C. After fixation, fish were washed 3 times in PBS pH7.4, and 1 time 1 hour in PBS pH6.0 at 4°C before incubation in the SA-β-gal staining solution (5 mM potassium ferrocyanide, 5 mM potassium ferricyanide, 2 mM MgCl_2_, 1 mg/mL X-gal in PBS pH6.0) overnight at 37°C. The same procedure was used for embryos fixed for 2 hours in 4% paraformaldehyde with PBS at room temperature. For adult zebrafish analyses, the trunk area for colorimetric quantification was chosen by selection of the area between the operculum and the dorsal and anal fins as described by Kishi et al. [39].

### Quantification of spinal curvature in zebrafish

Adult zebrafish were anaesthetized with MS-222 (0.168 mg/mL) and photographed with a digital camera. Using Image-J (NIH Image), a body midline was drawn and measured on digital images between the tip of the caudal peduncle and the center of the occipital orbit (“b” in S1 Fig). A perpendicular line was then drawn from the midline to the apex of the fish body (“a” in S1 Fig). The spinal curvature score was defined as the ratio of the length of the perpendicular line relative to the length of the fish (caudal peduncle to occipital orbit).

## Results

The zebrafish genome encodes six Psc family members [40]. These genes include *pcgf1* and *pcgf6* the orthologs of the *PCGF1* and *PCGF6* genes, respectively. In addition, following the whole-genome duplication that occurred in the teleost lineage after it splits from the tetrapod lineage, two duplicated ohnologs have been retained in the zebrafish genome for *BMI1* (*bmi1a* and *bmi1b*) and for *PCGF5* (*pcgf5a* and *pcgf5b*). Finally, *PCGF2* and *PCGF3* do not have corresponding homologs in zebrafish [40].

### Pcgf family member expression during zebrafish development

**Fig 1:**
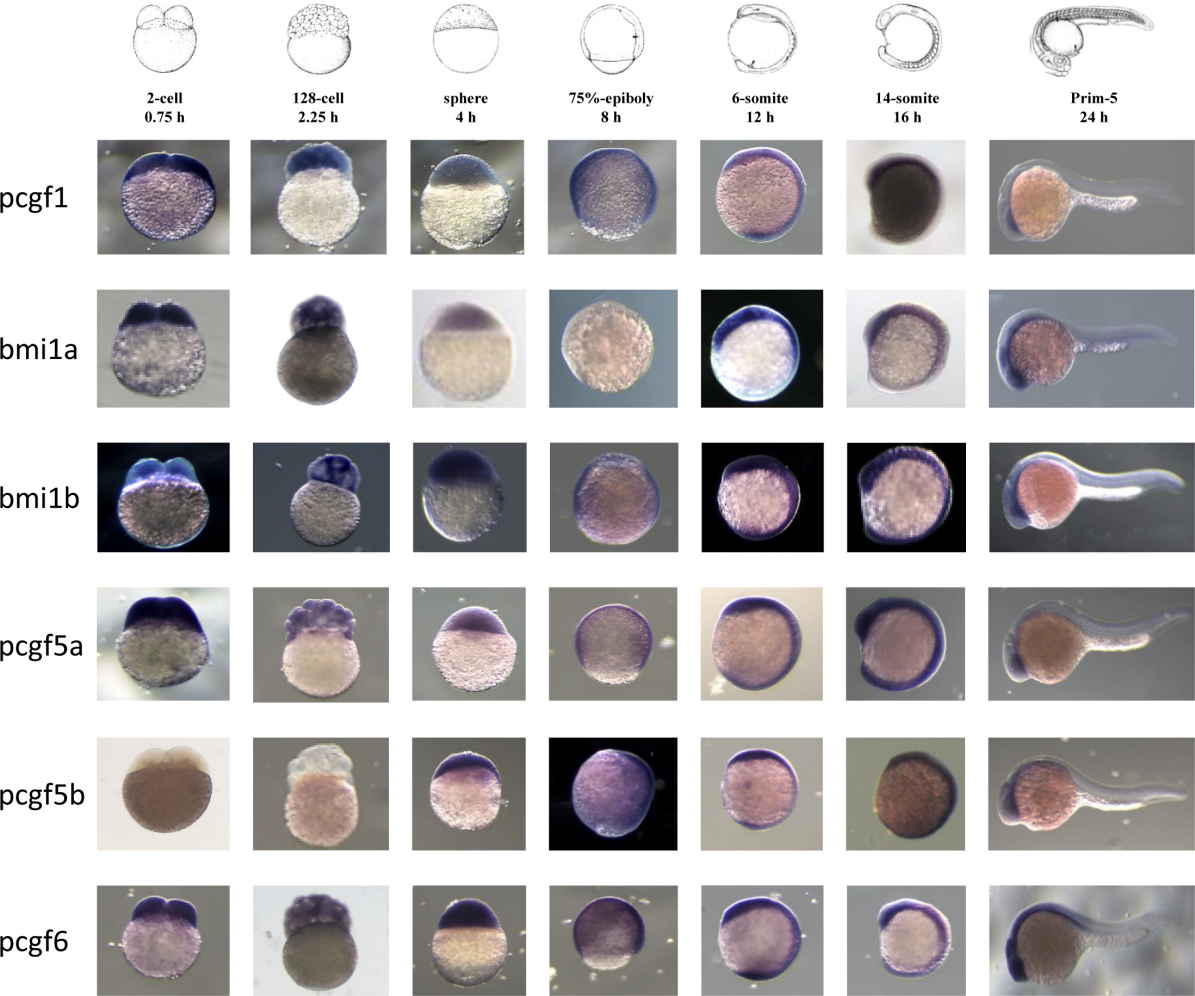
Expression of Psc orthologs during zebrafish development. Whole-mount *in situ* analyses of *pcgf1*, *bmi1a*, *bmi1b*, *pcgf5a*, *pcgf5b* and *pcgf6* zebrafish genes at 0.75, 2.25, 4, 8, 12, 16 and 24 hpf were representatively shown.

Zygotic transcription in zebrafish starts at about cell cycle 10–13 (3–4 hpf) termed midblastula transition (MBT). Before this stage, all developmental processes depend on maternally deposited gene products [41, 42]. We examined the expression patterns of the six Psc family members before the MBT (0.75 and 2.25 hpf), as well as at later zebrafish developmental stages (4, 8, 12, 16 and 24 hpf) (Fig 1). Whole-mount *in situ* hybridization revealed that most of the *pcgf* family gene members are ubiquitously expressed during zebrafish development. However, the retained ohnologs in the zebrafish genome showed distinct expression patterns. In contrast to the *bmi1b* transcripts, maternally deposited *bmi1a* transcripts significantly decrease at developmental stages around 4 hpf. Indeed, at 8 hpf, *bmi1a* transcripts are not detected by whole-mount *in situ* hybridization while *bmi1b* mRNAs are still present. Using GFP sensors [43–45] that contain the coding sequence of GFP and the *bmi1a* 3’UTR or *bmi1b* 3’UTR, we propose that determinants responsible for this maternal *bmi1a* mRNA decay may be encoded by the 3’UTR (S2 Fig). Interestingly, the *bmi1a* 3’UTR contains a putative binding site for the microRNA-430c (miR-430c) which has been shown to be involved in maternal mRNA decay during zygotic stages [43, 44]. There are also two onhologs for *pcgf5*; the *pcgf5a* transcript is present at all developmental stages, whereas *pcfg5b* expression starts only after the MBT. Notably, *pcgf5b* is the only pcgf family member which is not maternally expressed.

### Pcgf family member expression during zebrafish fin regeneration

The zebrafish caudal fin provides a powerful model to study complex tissue regeneration and bone repair because it is easily accessible and not essential for survival [46, 47]. Upon amputation, the caudal fin is fully restored after approximately 2 weeks. This type of regeneration, called epimorphic regeneration, involves the formation of a blastema, a population of proliferating progenitor cells that arise from dedifferentiation of mesenchymal cells in the stump. Continuous proliferation in the blastema causes structure outgrowth by providing new cells that differentiate into the different mesenchymal cell types needed to rebuild the lost fin part. This regeneration process requires a precise coordination of cell proliferation, cell differentiation, morphogenesis, and patterning. A number of reports demonstrated that epigenetic factors are involved and modulated in zebrafish caudal fin regeneration [48–50].

**Fig 2:**
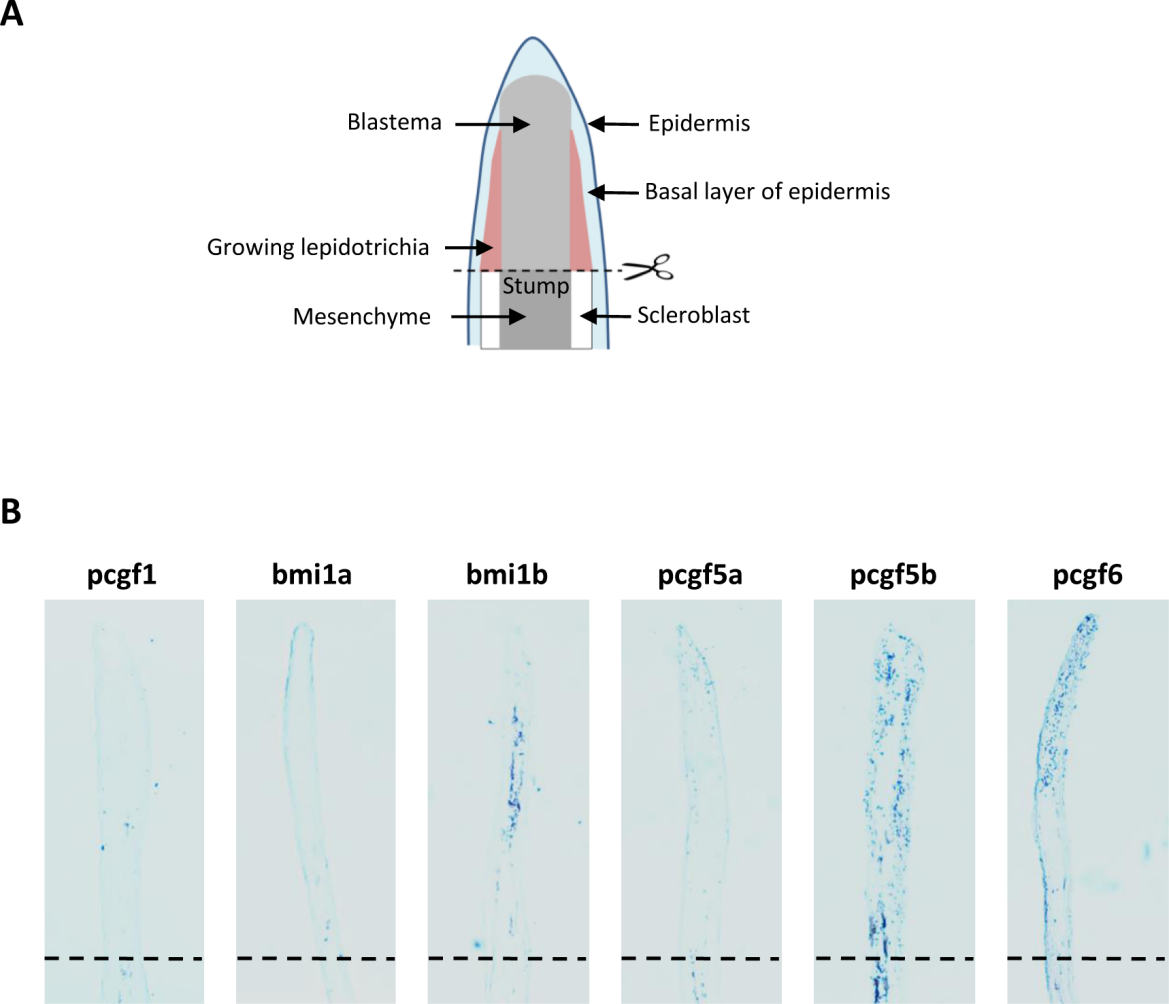
**Expression of the Pcgf family member during caudal fin regeneration** (**A**) Schematic representation of the regenerating caudal fin structures. (**B**) *In situ* hybridization with *pcgf1*, *bmi1a*, *bmi1b*, *pcgf5a*, *pcgf5b* and *pcgf6* RNA anti-sense probes on sections of regenerating caudal fins from 6 month-old zebrafish at 4 days post-amputation (dpa). Dashed lines indicate the amputation plane.

We then examined the expression of all Pcgf family members in regenerating caudal fin at 4 days post-amputation (dpa) by *in situ* hybridization on regenerating caudal fin sections (Fig 2). No *pcgf1* signal was detected in injured or regenerating fins at 4 dpa. Similarly, *pcgf5a* is not expressed in the regenerating tissues whereas a signal is observed in the stump. A strong *bmi1b* signal is observed in the medial region of the growing structures while *bmi1a* expression is restricted to the epidermis. A robust *pcgf5b* signal is also found in the growing lepidotrichia and in the blastema. Finally, *pcgf6* is expressed in the blastema of the regenerating fins. Thus, it appears that pcgf family members are diversely expressed in the regenerating caudal fin suggesting that they might contribute differently to the regeneration process by differential cellular distribution.

### TALEN-mediated *pcgf1* inactivation in zebrafish

**Fig 3:**
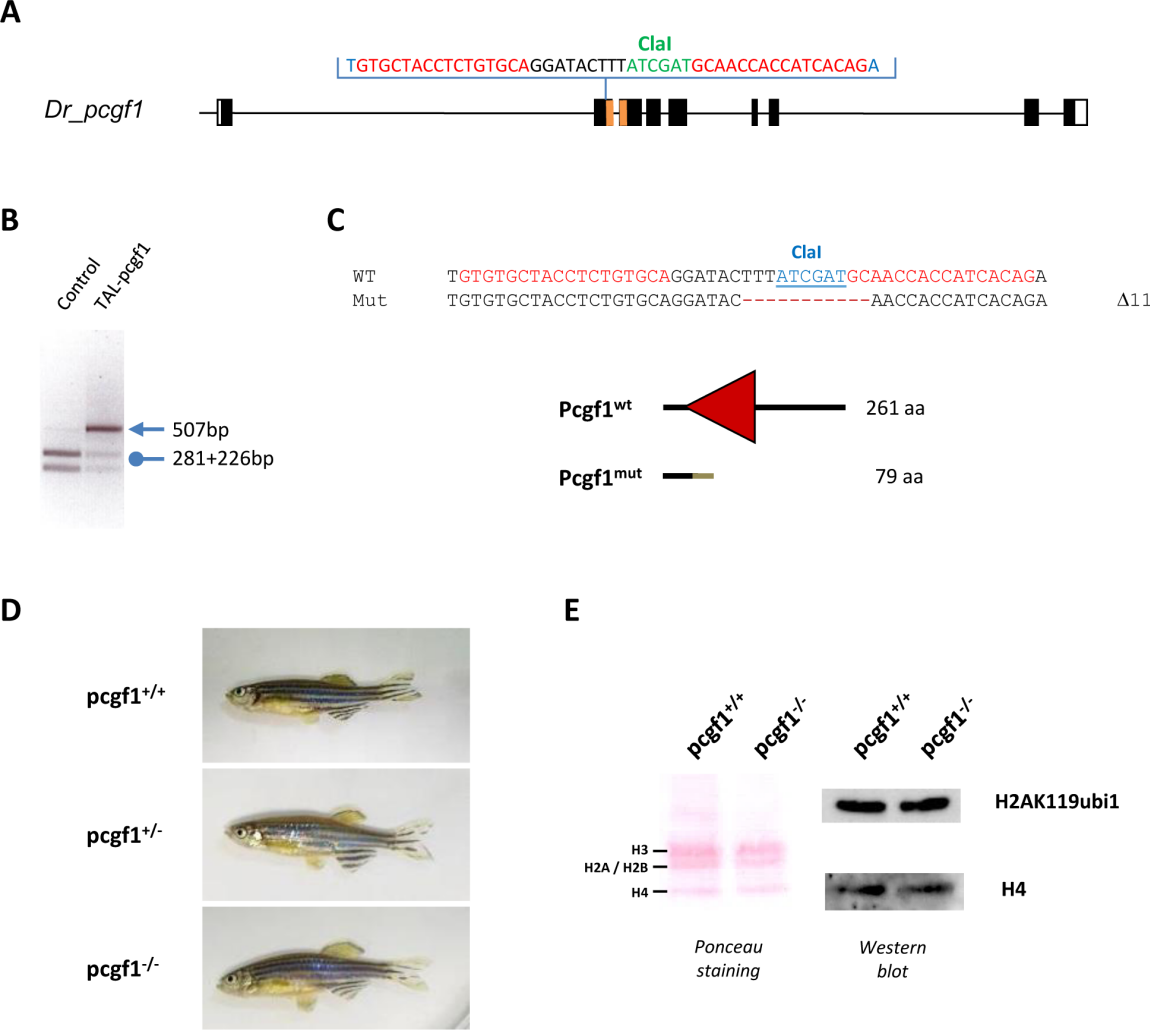
Generation of a pcgf1 mutant line using the TALEN technology. (**A**) Schematic representation of the genomic structure of the *pcgf1* gene, with coding and untranslated exon depicted as solid and open boxes, respectively. The exonic regions coding for the conserved RING finger motif are shown in orange. The location of the *pcgf1* TALEN in exon 2 is indicated. The *pcgf1* TALEN target sequence with Left and Right TALEN binding sites in red is shown. The ClaI restriction site is indicated in green. (**B**) Identification of mutant embryos using a diagnostic restriction. Genomic DNA was prepared from an uninjected (Control) and a *pcgf1* TALEN injected (TAL-pcgf1) embryo. The TALEN targeted DNA region is amplified by PCR and subjected to ClaI digestion. The TAL-pcgf1 injected embryo contains undigested material (arrow at 507 bp), indicating that the ClaI diagnostic restriction site has been disrupted. (**C**) Sequence of the mutant allele compared to its wild-type counterpart. Dashes indicate deleted nucleotides. For the peptide sequence, the gray line indicates residues read out of frame prior to encountering a premature stop codon and the red triangle corresponds to the RING finger motif in the wild-type protein. Size of the predicted proteins is indicated. (**D**) Pictures of siblings from a *pcgf1*^*+/-*^ x *pcgf1*^*+/-*^ cross at 7 months. The genotype of the fish was established using the ClaI diagnostic restriction on genomic DNA obtained by fin clipping. (**E**) Global monoubiquitinylation of histone H2A is not affected in *pcgf1*^*-/-*^ mutants. Total histone from pools of 5 embryos at 7 dpf were extracted and analyzed by western blotting. The efficiency of the transfer on the membrane is verified by Ponceau staining (Left) before incubation with anti-H2AK119ub1 and anti-H4 antibodies (Right).

To gain further insights into the role of *pcgf1* in zebrafish, we generated *pcgf1* loss of function mutants using transcription activator-like effector (TALE) nucleases (TALENs). TALENs consist in the fusion of the endonuclease domain of FokI to engineered sequence-specific DNA-binding domains from TALEs in order to target the nuclease activity to defined genomic sequences. Once activated through dimerization, the FokI nuclease introduces a double strand DNA break that is repaired through the non-homologous end joining (NHEJ) repair pathway. This repairing process is error prone and will introduce mutations. Among the resulting mutations, several will lead to shifts in the open reading frame, impairing the protein function. TALENs were designed to target a region within the second exon of *pcgf1* in order to introduce a frame shift upstream of known functional domains, particularly the RING finger motif. Furthermore, the targeted region was chosen to contain a ClaI restriction site that could be used to screen for mutations and for genotyping purposes (Fig 3A). TALENs were assembled using the Golden Gate Cloning methodology [38] and *in vitro* transcribed mRNAs encoding each TALEN pair were injected into one-cell stage embryos. Genomic DNA was extracted from single embryos collected at 3 days after TALEN mRNA injection. PCR amplification of the targeted region, followed by ClaI digestion revealed the efficacy of the designed TALENs and of the use of the diagnostic restriction site as a genotyping strategy (Fig 3B).

When analyzed by restriction of genomic DNA at 3 days after TALEN mRNA injection, the mutation rate was about 93% (14 of 15 injected embryos tested). Then, we raised TALENs-injected embryos to establish an adult F0 founder population. To evaluate the efficiency of germ line transmission of the mutations, individual F0 fish carrying mutations were crossed to wild-type TU partners to obtain F1 offspring. Genomic DNA was isolated from individual F1 embryos from each F0 fish and analyzed by ClaI restriction. Embryos from 4 of 6 individual F0 fish were heterozygous mutants, demonstrating successful germ line transmission of the mutations. One mutation causes a deletion of 11 bp (Δ11) leading to a frame shifting of the coding sequence and appearance of a premature stop codon (Fig 3C). The pcgf1^Δ11^ allele codes for a predicted protein of 79 amino acids lacking all conserved protein domains (S3A Fig) and was selected to raise the mutant zebrafish line used for further phenotypic studies after outcross to wild-type TU fish.

Among siblings from heterozygous *pcgf1*^*+/-*^ crosses, we successfully identified males and females carrying the homozygous Δ11 mutation (*pcgf1*^*-/-*^) (Fig 3D). The homozygous mutants are viable and fertile allowing us to generate a *pcgf1*^*-/-*^ line and thus, to demonstrate that both maternal and zygotic *pcgf1* products are dispensable to zebrafish development. Since the absence of *pcgf1* products does not affect viability and fertility of zebrafish, all the experiments done on *pcgf1*^*-/-*^ mutants were then performed on zebrafish *pcgf1*^*-/-*^ homozygous lines in order to exclude any *pcgf1* maternal contribution. Whole-mount *in situ* hybridization showed *pcgf1* expression in the anterior part of the wild-type embryos at 24 hpf. By contrast, *pcgf1* mRNA was not detectable in homozygous *pcgf1*^*-/-*^ mutants (S3B Fig), suggesting that the *pcgf1* mutant mRNA is degraded via nonsense-mediated decay. Since PCGF1 has been shown to promote monoubiquitinylation of histone H2A *in vitro* and in cells in culture [51], we next evaluated the monoubiquitinylation status of histone H2A in our *pcgf1*^*-/-*^ line. Total histones were extracted from mutant and wild-type embryos at 7 dpf and monubiquitinylation of histone H2A was analyzed by western blot using a specific anti-H2AK119ub1 antibody. Fig 3E reveals that global levels of monoubiquitinylated histone H2A are similar in *pcgf1*^*-/-*^ and *pcgf1*^*+/+*^ total embryos.

### Normal but retarded craniofacial development in *pcgf1* mutants

**Fig 4:**
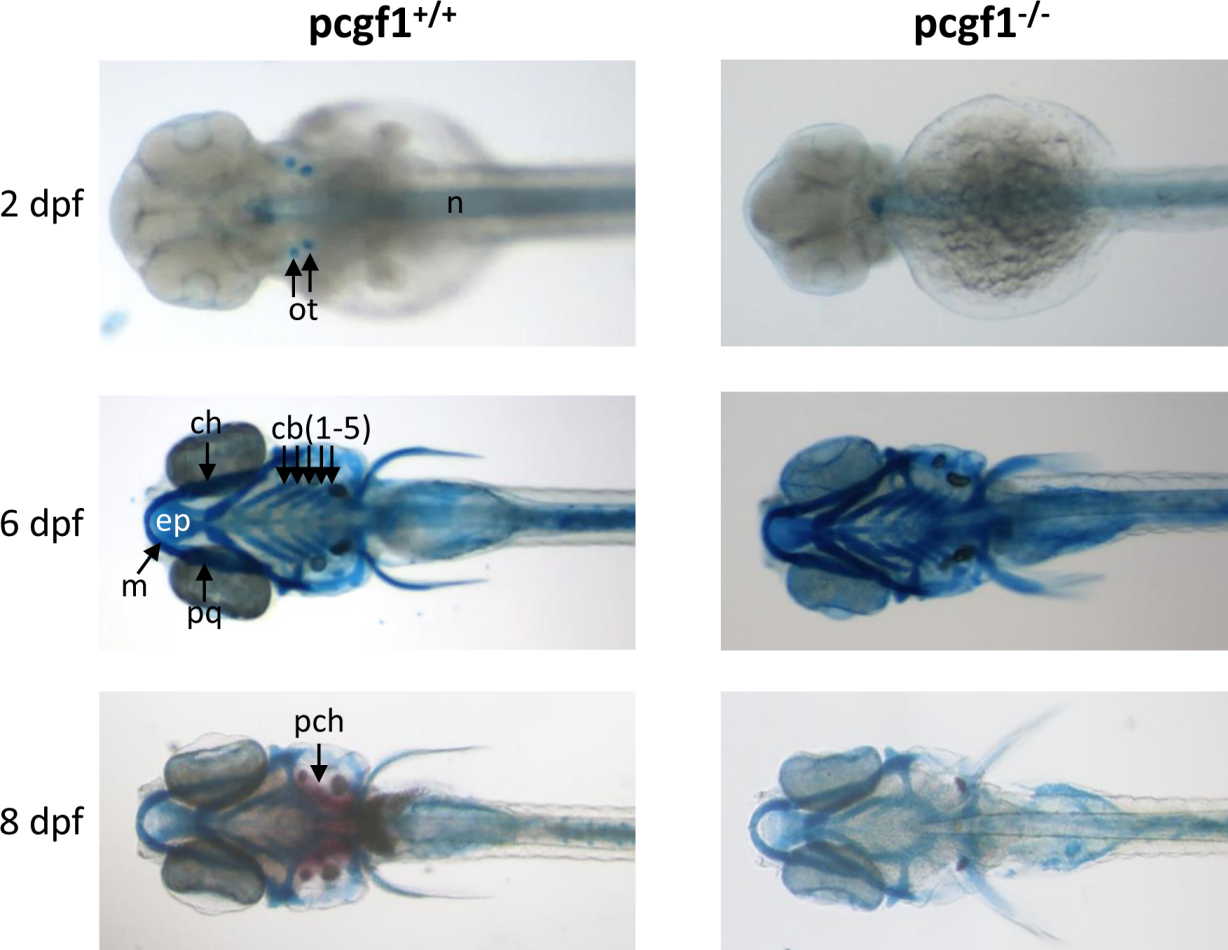
Development of cartilages and bones in *pcgf1*^*-/-*^ mutants. Alcian blue stained head cartilages and Alizarin red stained head bones of *pcgf1*^*+/+*^ (Left) and *pcgf1*^*-/-*^ mutant lines (Right) at the indicated developmental points. Note that ossification is visible at 8 dpf in the wild-type but not in the mutant. cb: ceratobranchials; ch: ceratohyal; ep: ethmoid plate; m: Meckel’s cartilage; n: notochord; ot: otoliths; pch: parachordal plate; pq: palatoquadrate.

Rnf2, the single E3 ubiquitin ligase of the zebrafish PRC1 complex, has been shown to be involved in zebrafish craniofacial development. Indeed, zebrafish *rnf2*^*-/-*^ mutants display a severe craniofacial phenotype, including an almost complete loss of all cranial cartilage, bone and musculature [52]. To investigate whether Pcgf1 function could also contribute to craniofacial development, *pcgf1*^*-/-*^ developing mutants were stained with Alcian blue and Alizarin red to visualize cartilage and bone formation, respectively [53] (Fig 4). At 2 dpf, Alcian blue stains the otoliths of wild-type fish, whereas these structures are not yet apparent in *pcgf1*^*-/-*^ mutant. At 6 dpf, cranial cartilages appear to be similar in wild-type and mutant fish. Ossification of the parachordal plate starts to be visible by Alizarin red staining in wild-type larvae at 8 dpf, but not in *pcgf1*^*-/-*^ larvae. This lack of Alizarin red staining in mutants reflects a delay, but not a defect, in the ossification process, since bones look normal at later developmental stages (S4 Fig).

**Fig 5:**
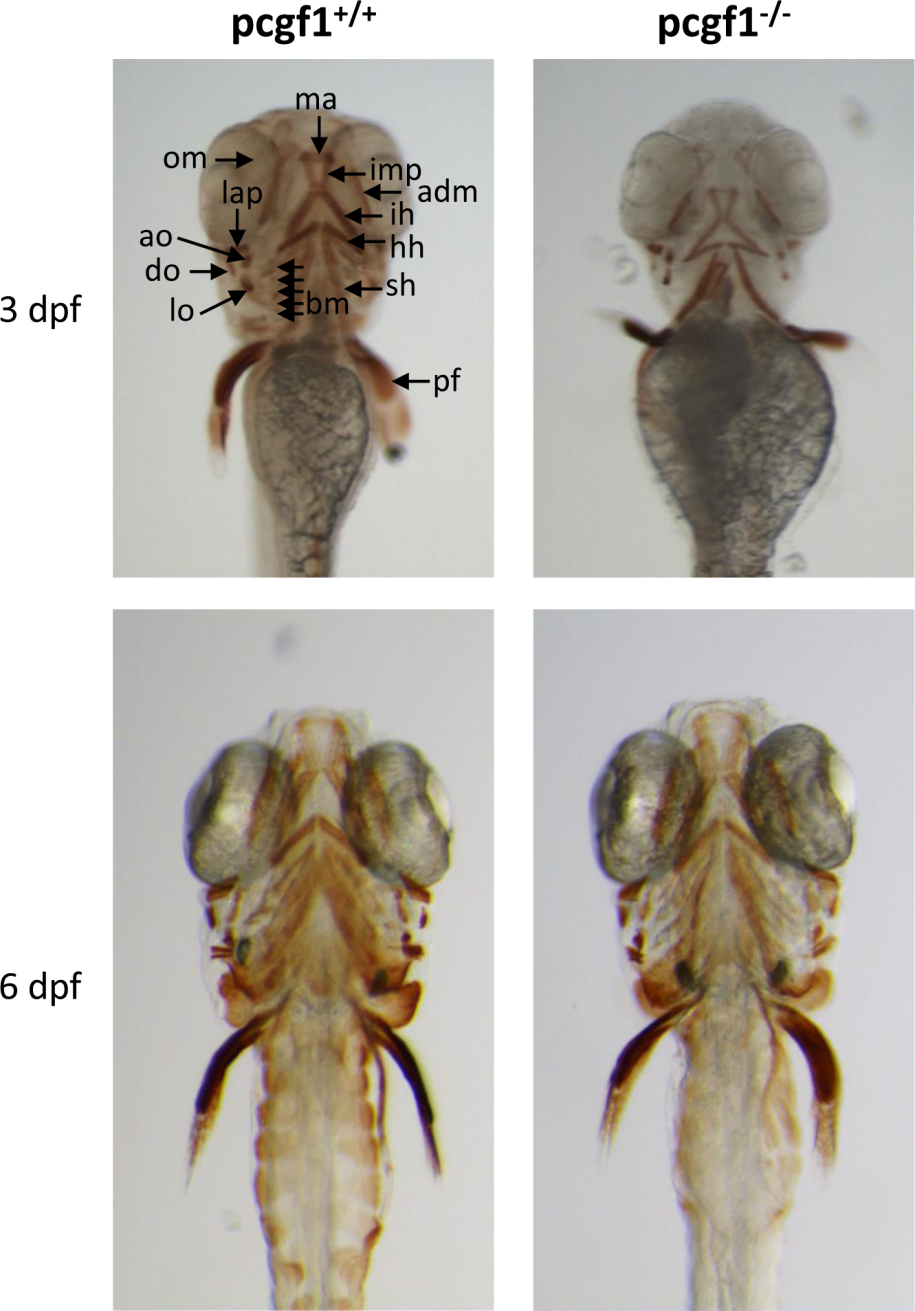
Cranial musculature in *pcgf1*^*-/-*^ mutants. Cranial musculature revealed by immunocytochemistry using the anti-myosin MF20 antibody shows that muscle development is delayed at 3 dpf but normal at 6 dpf in *pcgf1*^*-/-*^ larvae. adm: adductor mandibulae; ah: adductor hyomandibulae; ao: adductor operculi; bm: branchial musculatur; do: dilatator operculi; h: heart; hh:hyohyoideus; ih: interhyoideus; ima: intermandibularis anterior; imp: intermandibularis posterior; lap: levator arcus palatini; lo: levator operculi; om: occular muscle; pf: pectoral fin; sh: sternohyoideus.

An antibody that recognizes the myosin heavy chain of vertebrate striated muscle was used to analyze muscle development in *pcgf1*^*-/-*^ mutants (Fig 5). At 3 dpf, *pcgf1*^*-/-*^ larvae present a retarded cranial musculature. However, craniofacial musculature appears normal in *pcgf1*^*-/-*^ mutants at 6 dpf.

Taken together, our results indicate that in contrast to *rnf2* mutants, *pcgf1* mutants present a normal but delayed craniofacial development.

### Reduced growth of *pcgf1* mutants at early developmental stages

**Fig 6:**
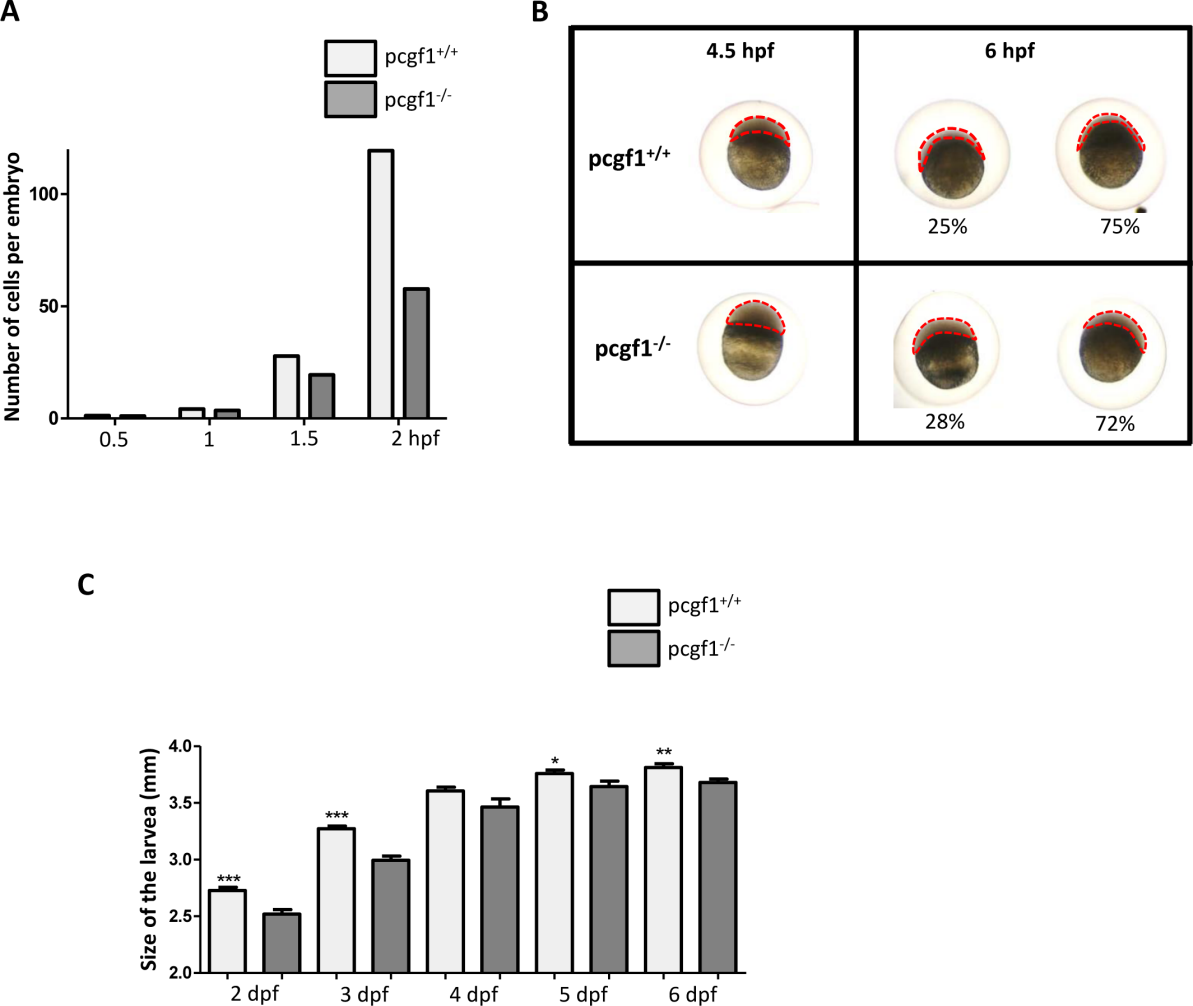
Growth of *pcgf1*^*-/-*^ mutants at early developmental stages. (**A**) Comparison of *pcgf1*^*+/+*^ and *pcgf1*^*-/-*^ embryo development during the cleavage period. Embryos from wild-type and mutant lines were fixed at 0.5, 1, 1.5 and 2 hpf, then 1-, 2-, 4-, 8-, 16-, 32-, 64-, 128- and 256-cell embryos were then counted, and results are expressed as the number of cells per embryo; n > 100 embryos per time point for each genotype. (**B**) Comparison of *pcgf1*^*+/+*^ and *pcgf1*^*-/-*^ embryo development at blastula and gastrula periods. Embryos were fixed at 4.5 and 6 hpf and representative embryos are shown. At 6 hpf embryos can be classified into 2 classes and the proportion of embryos of each group is indicated; n > 100 embryos. (**C**) Comparison of the size from head to tail, of fixed *pcgf1*^*+/+*^ and *pcgf1*^*-/-*^ larvae at 2, 3, 4, 5 and 6 dpf; Error bars indicate ± SD. Statistical significance was assessed by Student t-test analysis and significance expressed as the value of p (*, p < 0.1; **, p < 0.01, ***, p < 0.001). Measurements on embryos and larvae were done on 3 independent experiments using materials from different (3 to 6) layings.

We observed that cranial cartilage and muscle development was retarded in *pcgf1*^*-/-*^ mutants. To investigate whether development was also delayed at early stages, we measured zebrafish growth during the first 6 days post-fertilization (Fig 6). Comparison of *pcgf1*^*+/+*^ and *pcgf1*^*-/-*^ embryo development during the cleavage period revealed that mutant embryos develop at slightly lower rates (Fig 6A). During the blastula period, *pcgf1*^*-/-*^ mutants are also retarded compared to wild-type embryos (Fig 6B). At 4.5 hpf, *pcgf1*^*-/-*^ mutants are at the transition between the high and oblong stages, whereas *pcgf1*^*+/+*^ embryos reach the dome stage. Similarly, at 6 hpf during the gastrula period, the epiboly is more advanced in wild-type embryos in comparison to their *pcgf1*^*-/-*^ counterparts. Altogether, these results indicate that the delayed developmental growth observed in *pcgf1*^*-/-*^ embryos takes place at the very early developmental stages. At larval stages between 2 and 3 dpf, *pcgf1*^*-/-*^ mutants are still significantly smaller than wild-type larvae. Then, the difference in size between wild-type and mutant larvae progressively decreases till 6 dpf (Fig 6C). Later, once exogenous feeding begins, variability in size increases between larvae with the same genotype and *pcgf1*^*+/+*^ and *pcgf1*^*-/-*^ larvae cannot be distinguished based on their respective size.

**Fig 7:**
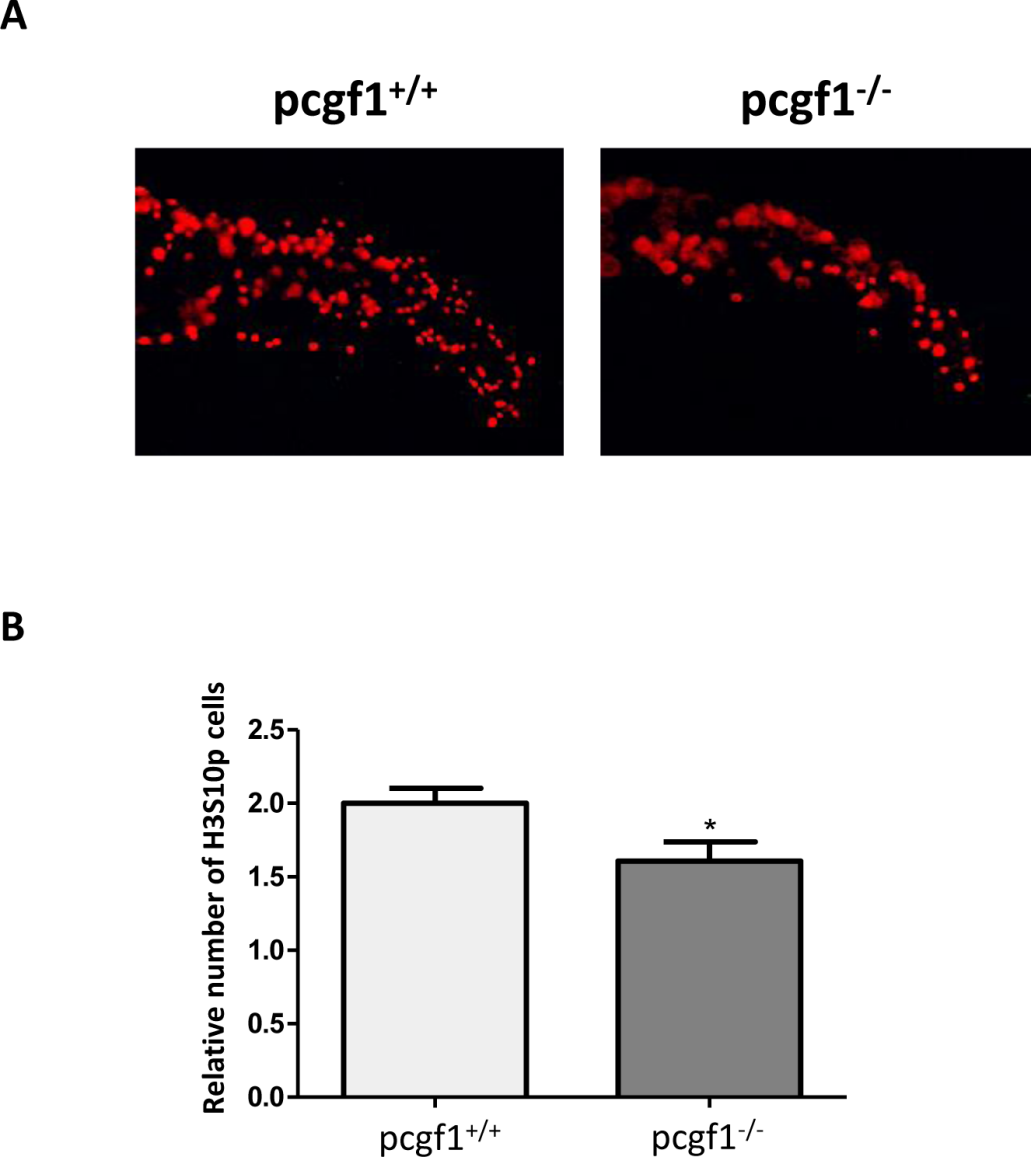
Cell proliferation measured by whole-mount immunohistochemistry using an anti-phosphohistone H3 antibody. (**A**) Antibody staining against phosphorylated histone H3 (anti-H3S10p) in *pcgf1*^*+/+*^ and *pcgf1*^*-/-*^ 24 hpf embryos. The caudal fin fold region of representative embryos is shown. (**B**) Quantification of H3S10p spots in the caudal fin fold expressed per unit of surface since *pcgf1*^*+/+*^ and *pcgf1*^*-/-*^ embryos have different sizes at this developmental stage. Error bars indicate ± SD. Statistical significance was assessed by Student t-test analysis and significance expressed as the value of p and * indicates p < 0.01.

In order to investigate whether the retarded growth of *pcgf1*^*-/-*^ mutants at early developmental stages could be due to reduced cell proliferation, we applied whole-mount immunohistochemistry with the mitotic marker phosphohistone H3 since phosphorylation of histone H3 at serine 10 constitutes a good marker for cell proliferation in zebrafish embryos [54, 55]. Fig 7 shows that *pcgf1*^*-/-*^ embryos harbor less mitotic cells than wild-type embryos at 24 hpf. Moreover apoptosis studies by Acridine orange staining and analysis of cellular senescence by senescence-associated β-galactosidase assays were not able to detect significant differences between *pcgf1*^*+/+*^ and *pcgf1*^*-/-*^ embryos at this stage (S5 Fig). Taken together, these results indicate that the reduced growth of *pcgf1*^*-/-*^ embryos is mainly due to reduced cell proliferation, but not to an increase in apoptosis or senescence.

### *pcgf1* mutants display an early aging phenotype

**Fig 8:**
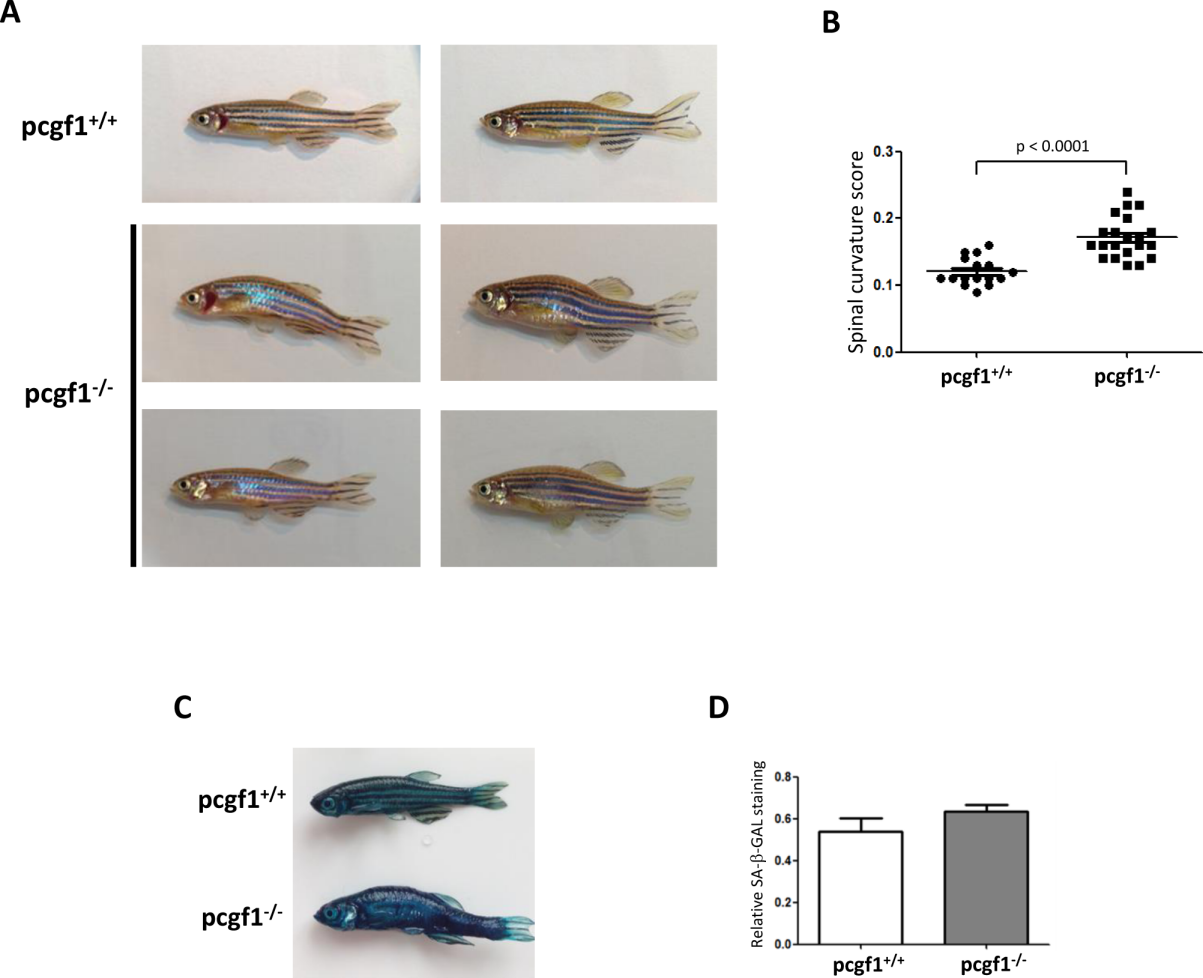
Phenotype of 12 month-old *pcgf1*^*-/-*^ mutants. (**A**) Pictures of 12 month-old *pcgf1*^*+/+*^ and *pcgf1*^*-/-*^ fish. Note that both male and female *pcgf1*^*-/-*^ fish exhibit mild spinal curvature. (**B**) Measurements of the spinal curvature score in 12 month-old fish from the *pcgf1*^*+/+*^ and *pcgf1*^*-/-*^ populations. Statistical significance was assessed by Student t-test analysis and significance expressed as the value of p. (**C**) Senescence-associated β-galactosidase (SA-β-Gal) activity in 12 month-old zebrafish. Whole body staining of SA-β-Gal activity in *pcgf1*^*+/+*^ and *pcgf1*^*-/-*^ fish harboring a spinal curvature are compared. (**D**) Quantification of SA-β-Gal activity in *pcgf1*^*+/+*^ and *pcgf1*^*-/-*^ fish. Blue pixels obtained by whole body staining of SA-β-Gal activity were quantified in the trunk area between the operculum and the dorsal and anal fins by Adobe Photoshop and plotted using Prism (GraphPad). Error bars indicate ± SD.

Although *pcgf1*^*-/-*^ zebrafish are viable and fertile, we observed that approximately 35% of mutant exhibit a spinal curvature at the age of 12 months (Fig 8A and 8B). This spinal curvature phenotype is a common morphological aging manifestation in zebrafish [56, 57]. Such a morphological alteration has not been observed in our wild-type fish collection at this age, but was also found in some heterozygous *pcgf1*^*+/-*^ zebrafish as well as in *pcgf1*^*-/-*^ mutants at the age of 6 months (S6 Fig). Since, we could not find abnormalities in the skeleton development in *pcgf1*^*-/-*^ fish (data not shown and S4 Fig), we assume that the spinal curvature observed in mutant fish reflect a premature aging phenotype. In addition, 12 month-old *pcgf1*^*-/-*^ zebrafish exhibit a stronger senescence-associated β-galactosidase (SA-β-Gal) activity when compared to control individuals (Fig 8C and 8D). Then, these results suggest that loss of *pcgf1* function may favor accelerated aging in zebrafish.

## Discussion

Polycomb Group (PcG) protein complexes regulate gene expression by silencing target genes through chromatin structure modulation. Among these complexes, canonical PRC1 core complex is formed by a combination of 4 proteins, CBX, PCGF, PHC, and E3-ligase protein that catalyzes the monoubiquitinylation of histone H2A at lysine 119 (H2AK119ub1). In mammals, these proteins have multiple orthologs, including five CBX (CBX2, 4, 6, 7 and 8), six PCGF (BMI1, PCGF1, 2, 3, 5 and 6), three PHC (PHC1, 2 and 3) and two E3-ligases (RING1 and RNF2). Different associations of these various family members generate multiple PRC1 variant complexes with potentially distinct biological functions.

Here, we address the question of the redundancy of PCGF family members in zebrafish. Like in mammals, the zebrafish genome encodes five PCGF family members. These members are Pcgf1, the ortholog of human PCGF1, two orthologs for BMI1, named Bmi1a and Bmi1b, two PCGF5 orthologs, Pcgf5a and Pcgf5b, and Pcgf6, the ortholog of PCGF6 [40].

Whole-mount *in situ* hybridization showed that expression of the zebrafish *pcgf* genes is not spatially restricted and all genes but not *pcgf5b*, are maternally expressed. It is also remarkable that the couples of onhologs which appeared after the genome duplication that occurred in the teleost lineage are characterized by distinct developmental expression profiles. In this regard, *pcgf5b* is not maternally expressed whereas *pcgf5a* is. Similarly, maternal *bmi1a* transcript levels decrease at about 4 hpf and are not detected anymore by whole-mount *in situ* hybridization at 8 hpf, whereas *bmi1b* transcripts remain abundant at all developmental stages. Zebrafish microRNA-430 (miR-430) family is highly expressed during early development. It targets hundreds of mRNAs and is required for clearance of hundred maternal transcripts during zygotic stages [43, 44]. Interestingly, we identified a putative miR-430-binding site within the *bmi1a* 3’UTR and showed that this *bmi1a* 3’UTR, but not the *bmi1b* 3’UTR, contains determinants responsible for the reduction of the GFP activity using a GFP sensor assay. Thus, this differential post-transcriptional regulation of the two *bmi1* onhologs potentially by the micro RNA miR-430, illustrates that various zebrafish *pcgf* genes are subjected to distinct expression controls during development. A differential expression of the *pcgf* genes is also found during the caudal fin regeneration. *pcgf1* and *pcgf5a* transcripts are not detected in regenerating caudal fin sections at 4 dpa by *in situ* hybridization, but *bmi1a* expression is found in the epidermis, *bmi1b* in medial structures, whereas *pcgf5b* and *pcgf6* are expressed in larger regions of regenerating fins. The fact that all the *pcgf* genes are not expressed in the same regenerating fin regions indicates that distinct PRC1 complexes, in terms of Pcgf subunit composition, do not necessarily co-exist in the same cells. Pcgf1- and Pcgf5a-containing PRC1 complexes are for instance absent from the regenerating tissues after amputation of the caudal fin. Our observations show that expression of all *pcgf* gene members and concomitant presence of all PRC1 complex combinations is not required for viability of all cell types.

To investigate the function of Pcgf1, we generate a *pcfg1*-loss of function zebrafish line using the TALEN technology. Homozygous *pcgf1*^*-/-*^ fish are viable and fertile indicating that both maternal and zygotic pcgf1 products are dispensable to zebrafish development.

As part of the PRC1 complex, PCGF1 has been shown to enhance monoubiquitinylation of H2A both *in vitro* and in cells in culture [51]. Indeed, knock down of PCGF1 by siRNAs reduces H2A ubiquitinylation levels in HeLa cells [51]. In contrast, we show here that *pcgf1* inactivation in zebrafish does not affect global levels of monoubiquitinylated histone H2A in embryos at 7 dpf, suggesting that Pcgf1-containing PRC1 complexes might contribute to a minor part of total H2A ubiquitinylation. In *pcgf1*^*-/-*^ zebrafish, the absence of Pcgf1-containing PRC1 complex activity could be compensated by the presence of other PRC1 complexes. The normal H2A ubiquitinylation levels in *pcgf1*^*-/-*^ fish might also explain why these mutants do not recapitulate the phenotypes observed in zebrafish harboring a mutation in the rnf2 gene coding for the E3-ligase activity of the PRC1 complex [52, 58]. However, we cannot exclude a possible contribution of Pcgf1 to PRC1 activity in a rare subpopulation such as certain progenitors or stem cells. In such a case, even if Pcgf1 is specifically required for PRC1 function, it is likely that inactivation of *pcgf1* function could not affect global changes in H2A ubiquitinylation due to the limitation in cell number.

The analysis of embryonic growth at the cleavage, blastula and gastrula periods revealed that *pcgf1*^*-/-*^ mutants grow at slightly lower rates. We showed that this reduced growth correlates with cell proliferation decrease, but not with an increase in cell senescence or apoptosis. Also, at larval stages, *pcgf1*^*-/-*^ mutants are still significantly smaller than wild-type fish till 6 dpf. After exogenous feeding starts, *pcgf1*^*+/+*^ and *pcgf1*^*-/-*^ larvae cannot be distinguished based on their size, but mutants still show a retarded but normal, cranial cartilage, bone and musculature development.

In addition to a reduce growth rate and developmental delays at early stages, we observed an unusual number of *pcgf1*^*-/-*^ homozygous fish exhibiting a spinal curvature before the age of 12 months. This curvature is one common morphological aging manifestation in zebrafish [56, 57], suggesting that Pcgf1 function may prevent premature aging. However, the spinal curvature phenotype is not fully penetrant and found in about 35% of mutant fish at 12 months, indicating that additional factors contribute together with Pcgf1 to aging. Such a link between epigenetics and aging has also been reported for other PCGF family members. In particular, *Bmi1*-deficient mice exhibit postnatal defects in the self-renewal of adult stem cells and the aging-associated dysregulation of the Bmi1/p16^Ink4a^ pathway may account for failures in tissue repair in aging [59–62]. Interestingly, a genome-wide gain-of-function genetic screen in mouse embryonic stem (ES) cells also identifies Pcgf1 as a factor able to promote the expression of the ES cell pluripotency markers and able to partially rescue ES cell growth in the absence of leukemia inhibitory factor (LIF) [63]. Thus, we could speculate that reduced proliferation growth of certain progenitors and stem cells in *pcgf1*-deficient fish could impair both the growth at early developmental stages and later the tissue repair thus accelerating the aging phenotype appearance.

## Conclusions

In this work, we show that *pcgf* family gene members are differentially regulated during zebrafish development and during the process of caudal fin regeneration. Then, the different PRC1 complexes in terms of Pcgf subunit composition are not always present in the same tissues during development and caudal fin regeneration. We also offer the first vertebrate model lacking Pcgf1 function and reveal that this Polycomb Group protein is involved in cell proliferation during early zebrafish embryogenesis. Finally our zebrafish model may also highlight a link between epigenetics and aging.

## Supporting Information

S1 Fig Method of measurement of the spinal curvature score in zebrafish. A body midline is drawn and measured between the tip of the caudal peduncle and the center of the occipital orbit (b). A perpendicular line is then drawn from the midline to the apex of the fish body (a). The spinal curvature score is defined as the ratio of the length of the perpendicular line relative to the length of the fish (a/b).
(PDF)

S2 Fig The *bmi1a* 3’UTR reduces GFP expression levels in zebrafish embryos. (**A**) Comparison of *bmi1a* and *bmi1b* expression levels during zebrafish development analyzed by whole-mount *in situ* hybridization. Note that *bmi1a* transcripts are not detected at 8 hpf while *bmi1b* transcripts are present. (**B**) Organization of the *bmi1a* and *bmi1b* transcripts. A predicted base pairing between miR-430c and the *bmi1a* 3’UTR is shown. (**C**) Experimental set-up to test the effect of the *bmi1a* 3’UTR on GFP expression levels. A GFP reporter mRNA containing no 3’UTR (control), the *bmi1a* 3’UTR or the *bmi1b* 3’UTR is co-injected with control dsRed (RFP) mRNA into 1-cell stage embryos. (**D**) GFP reporter expression (green) and control dsRed expression (red) at 4–5 hpf monitor mRNA injection (left panels). GFP reporter expression and control dsRed expression at 28 hpf reveals that *bmi1a* 3’UTR reduces GFP expression levels (right panels). (**E**) Quantification of relative GFP expression levels. Error bars indicate ± SD; n > 15 embryos per experiment; Statistical significance was assessed by Student t-test analysis and significance expressed as the indicated p values.
(PDF)

S3 Fig Products of the *pcgf1*^*Δ11*^ allele. (**A**) Predicted protein encoded by the *pcgf1*^*Δ11*^ allele (Pcgf1^mut^) compared to the wild-type Pcgf1 protein. Peptides coding for the RING finger and the PCGF conserved motif are indicated in red and green, respectively [40]. (**B**) Whole-mount in situ analysis of *pcgf1* expression on *pcgf1*^*+/+*^ and *pcgf1*^*-/-*^ embryos at the prim-5 stage (about 24 hpf).
(PDF)

S4 Fig Skeletal development of *pcgf1*^*-/-*^ zebrafish mutants using Alcian blue-Alizarin red double staining. Details of the cartilage and bone structures at the caudal (**A**), dorsal and anal fins (**B**) show that skeletal structures are formed, calcified and normal at 21 dpf. ep, epural; hspu: haemal spine of preural; hy: hypural; nspu: neural spine of preural; opstc: opistural cartilage; phy: parhypural; adr: anal distal radial; apr: anal proximal radial; ddr: dorsal distal radials; dpr: dorsal proximal radial.
(PDF)

S5 Fig Analysis of apoptosis and senescence in *pcgf1*^*-/-*^ mutants at 24 hpf. (**A**) Apoptosis detection by Acridine orange staining of live embryos at 24 hpf. The caudal fin fold region of representative embryos is shown. (**B**) Senescence-associated β-galactosidase detection in 24 hpf embryos. Representative *pcgf1*^*+/+*^ and *pcgf1*^*-/-*^ embryos are shown.
(PDF)

S6 Fig Phenotype of 6 month-old *pcgf1*^*-/-*^ zebrafish. Example of 6 month-old *pcgf1*^*-/-*^ zebrafish harboring no (top), weak (middle) or more pronounced (bottom) spinal curvatures.
(PDF)
