# Supplementary figures and images for: The Polycomb Group Protein Pcgf1 Is Dispensable in Zebrafish but Involved in Early Growth and Aging
Source: PLoS One. 2016 Jul 21;11(7):e0158700. doi: 10.1371/journal.pone.0158700 (PMC4956247; doi:10.1371/journal.pone.0158700)

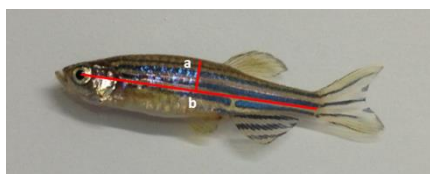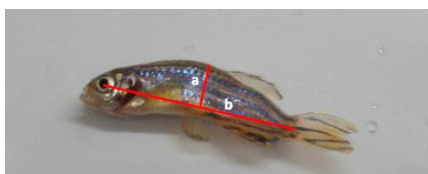

$$\text{Spinal curvature score} = \frac{a}{b}$$

Supplement: S1 Fig — A body midline is drawn and measured between the tip of the caudal peduncle and the center of the occipital orbit (b). A perpendicular line is then drawn from the midline to the apex of the fish body (a). The spinal curvature score is defined as the ratio of the length of the perpendicular line relative to the length of the fish (a/b). (PDF) [file pone.0158700.s001.pdf]

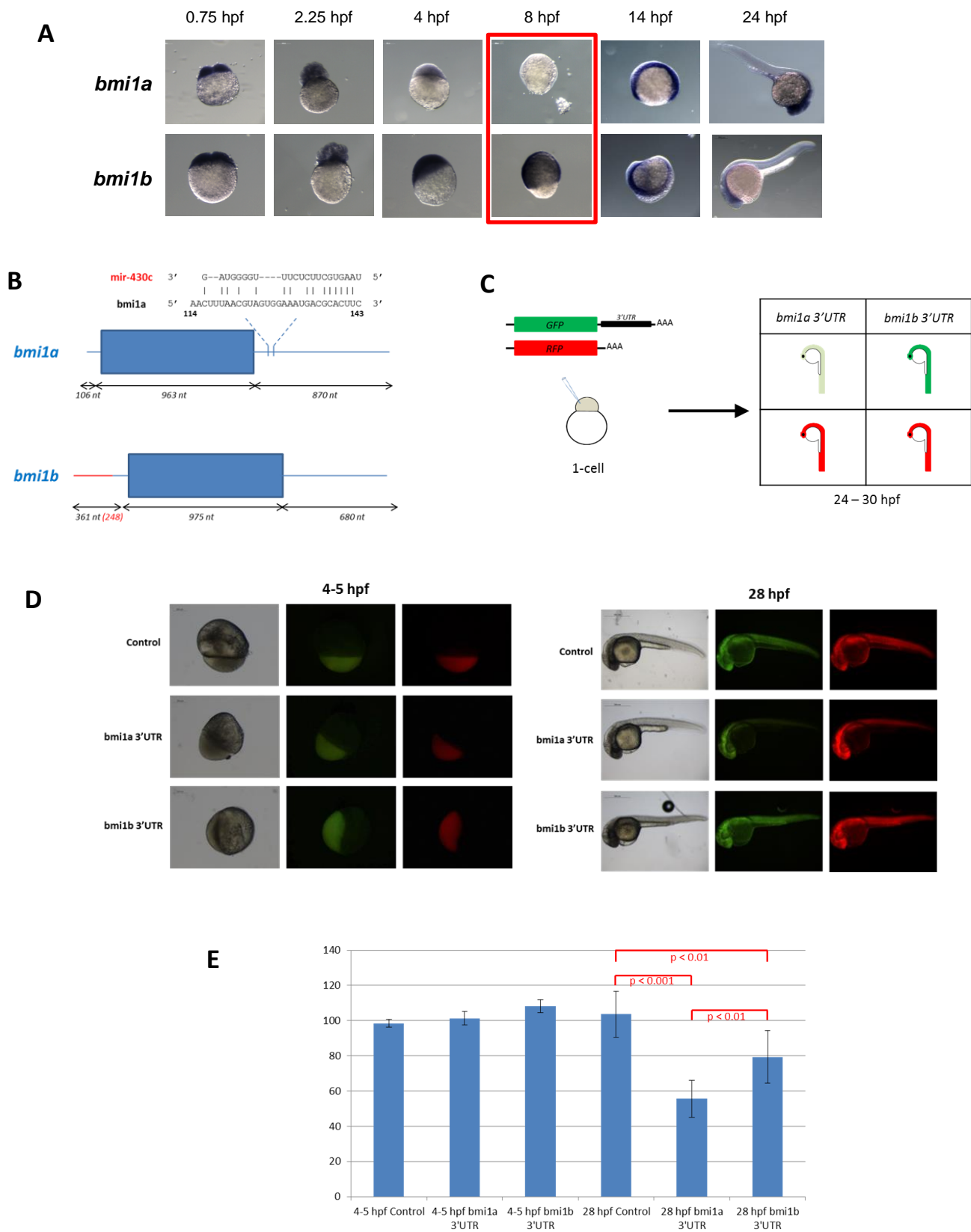

Supporting Information – S2 Fig

Supplement: S2 Fig — (A) Comparison of bmi1a and bmi1b expression levels during zebrafish development analyzed by whole-mount in situ hybridization. Note that bmi1a transcripts are not detected at 8 hpf while bmi1b transcripts are present. (B) Organization of the bmi1a and bmi1b transcripts. A predicted base pairing between miR-430c and the bmi1a 3’UTR is shown. (C) Experimental set-up to test the effect of the bmi1a 3’UTR on GFP expression levels. A GFP reporter mRNA containing no 3’UTR (control), the bmi1a 3’UTR or the bmi1b 3’UTR is co-injected with control dsRed (RFP) mRNA into 1-cell stage embryos. (D) GFP reporter expression (green) and control dsRed expression (red) at 4–5 hpf monitor mRNA injection (left panels). GFP reporter expression and control dsRed expression at 28 hpf reveals that bmi1a 3’UTR reduces GFP expression levels (right panels). (E) Quantification of relative GFP expression levels. Error bars indicate ± SD; n > 15 embryos per experiment; Statistical significance was assessed by Student t-test analysis and significance expressed as the indicated p values. (PDF) [file pone.0158700.s002.pdf]

**A**

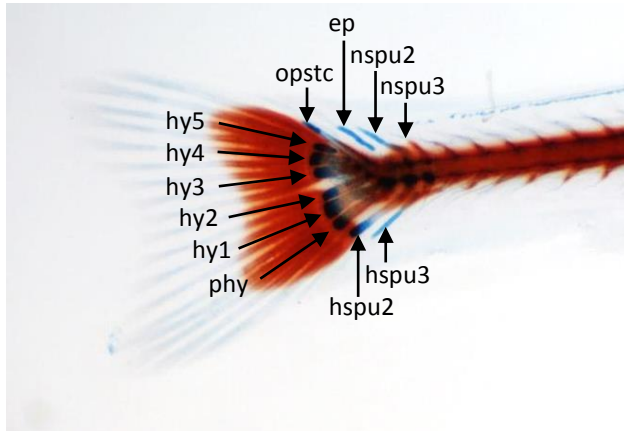

**B**

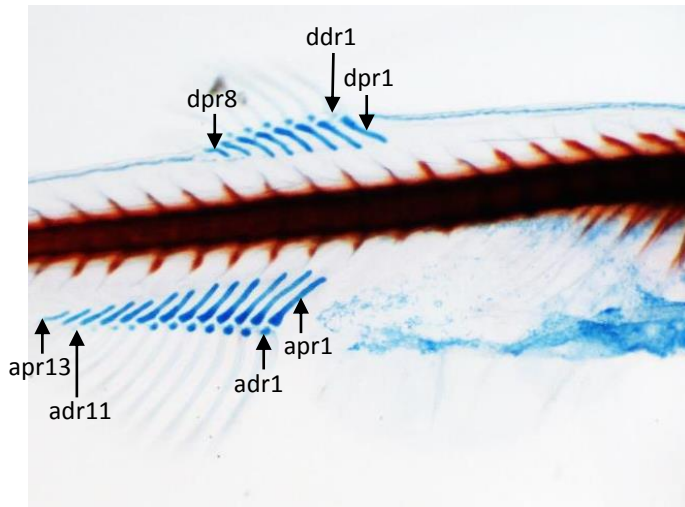

Supplement: S4 Fig — Details of the cartilage and bone structures at the caudal (A), dorsal and anal fins (B) show that skeletal structures are formed, calcified and normal at 21 dpf. ep, epural; hspu: haemal spine of preural; hy: hypural; nspu: neural spine of preural; opstc: opistural cartilage; phy: parhypural; adr: anal distal radial; apr: anal proximal radial; ddr: dorsal distal radials; dpr: dorsal proximal radial. (PDF) [file pone.0158700.s004.pdf]

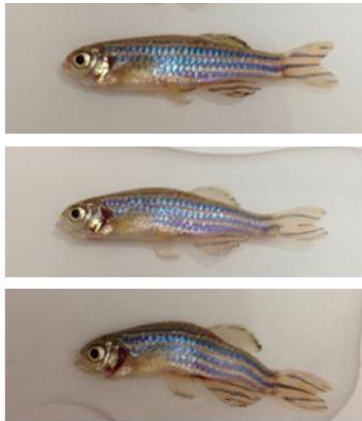

Supplement: S6 Fig — Example of 6 month-old pcgf1-/- zebrafish harboring no (top), weak (middle) or more pronounced (bottom) spinal curvatures. (PDF) [file pone.0158700.s006.pdf]
