# Supplementary material for: The Polycomb Group Protein Pcgf1 Is Dispensable in Zebrafish but Involved in Early Growth and Aging
Source: PLoS One. 2016 Jul 21;11(7):e0158700. doi: 10.1371/journal.pone.0158700 (PMC4956247; doi:10.1371/journal.pone.0158700)
Supplement: S3 Fig — (A) Predicted protein encoded by the pcgf1Δ11 allele (Pcgf1mut) compared to the wild-type Pcgf1 protein. Peptides coding for the RING finger and the PCGF conserved motif are indicated in red and green, respectively [40]. (B) Whole-mount in situ analysis of pcgf1 expression on pcgf1+/+ and pcgf1-/- embryos at the prim-5 stage (about 24 hpf). (PDF) [file pone.0158700.s003.pdf]

A

Pcgf1^{wt}

MAEQGPMAIAMRLRNQLQNVYKLDPLRNEEEVKIKIKDLNEHIV
CYLCAGYFIDATTITECLHTFCKSCIVKYLQTSKYCPMCNIKIH
ETQPLLNLKLDRVMQDIVYKLVPGLQESEDKRIKEFYQSRGLER
IIQPSGEESVPDNTGLPYTSFDHSKAHFYRYDEQVSLCLERQSS
SFSGKDKNKLTLQQKFVRCSVRAEVRHLRKVLCHRLNVEKHQVQ
MLFNNESLPDHMTMKRLWLSHWFGKAQPLVLHYTIKDKRTR*

Pcgf1^{mut}

MAEQGPMAIAMRLRNQLQNVYKLDPLRNEEEVKIKIKDLNEHIV
CYLCAGY↓NHHHRMSPYVLQELHCEVSPDQQVLSNV*

B

pcgf1$^{(+/+)}$

pcgf1$^{(-/-)}$

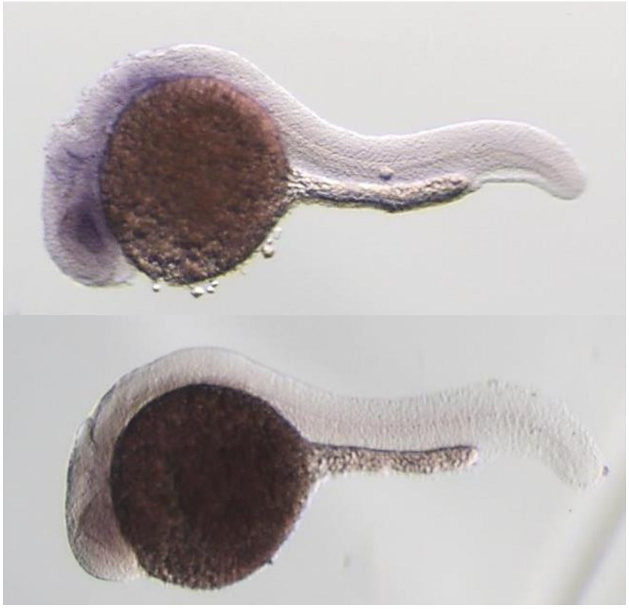

Supporting Information – S3 Fig
