# Supplementary material for: The Polycomb Group Protein Pcgf1 Is Dispensable in Zebrafish but Involved in Early Growth and Aging
Source: PLoS One. 2016 Jul 21;11(7):e0158700. doi: 10.1371/journal.pone.0158700 (PMC4956247; doi:10.1371/journal.pone.0158700)
Supplement: S5 Fig — (A) Apoptosis detection by Acridine orange staining of live embryos at 24 hpf. The caudal fin fold region of representative embryos is shown. (B) Senescence-associated β-galactosidase detection in 24 hpf embryos. Representative pcgf1+/+ and pcgf1-/- embryos are shown. (PDF) [file pone.0158700.s005.pdf]

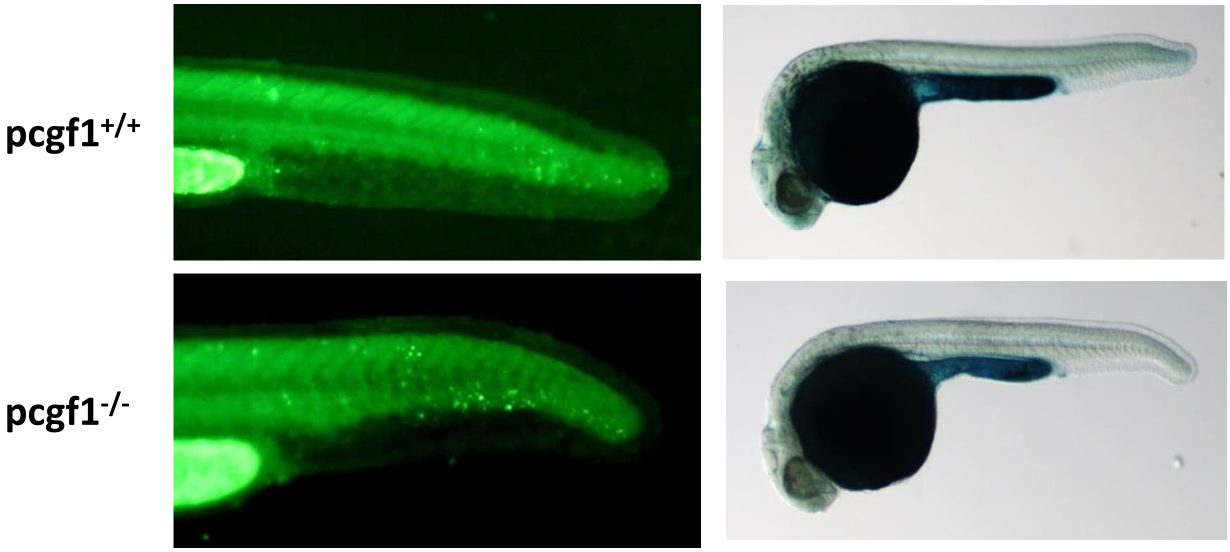

Apoptosis detection by acridine orange

Detection of senescence-associated β-galactosidase

Supporting Information – S5 Fig
